# Identifying a Kinase Network Regulating FGF14:Nav1.6 Complex Assembly Using Split-Luciferase Complementation

**DOI:** 10.1371/journal.pone.0117246

**Published:** 2015-02-06

**Authors:** Wei-Chun Hsu, Miroslav N. Nenov, Alexander Shavkunov, Neli Panova, Ming Zhan, Fernanda Laezza

**Affiliations:** 1 Department of Pharmacology and Toxicology, University of Texas Medical Branch, Galveston, Texas, United States of America; 2 M.D./Ph.D. Combined Degree Program, University of Texas Medical Branch, Galveston, Texas, United States of America; 3 Mitchell Center for Neurodegenerative Diseases, University of Texas Medical Branch, Galveston, Texas, United States of America; 4 Center for Biomedical Engineering, University of Texas Medical Branch, Galveston, Texas, United States of America; 5 Center for Addiction Research, University of Texas Medical Branch, Galveston, Texas, United States of America; 6 Department of Systems Medicine and Bioengineering, Weill Cornell Medical College, Methodist Hospital Research Institute, Houston, Texas, United States of America; University of Louisville, UNITED STATES

## Abstract

Kinases play fundamental roles in the brain. Through complex signaling pathways, kinases regulate the strength of protein:protein interactions (PPI) influencing cell cycle, signal transduction, and electrical activity of neurons. Changes induced by kinases on neuronal excitability, synaptic plasticity and brain connectivity are linked to complex brain disorders, but the molecular mechanisms underlying these cellular events remain for the most part elusive. To further our understanding of brain disease, new methods for rapidly surveying kinase pathways in the cellular context are needed. The bioluminescence-based luciferase complementation assay (LCA) is a powerful, versatile toolkit for the exploration of PPI. LCA relies on the complementation of two firefly luciferase protein fragments that are functionally reconstituted into the full luciferase enzyme by two interacting binding partners. Here, we applied LCA in live cells to assay 12 kinase pathways as regulators of the PPI complex formed by the voltage-gated sodium channel, Nav1.6, a transmembrane ion channel that elicits the action potential in neurons and mediates synaptic transmission, and its multivalent accessory protein, the fibroblast growth factor 14 (FGF14). Through extensive dose-dependent validations of structurally-diverse kinase inhibitors and hierarchical clustering, we identified the PI3K/Akt pathway, the cell-cycle regulator Wee1 kinase, and protein kinase C (PKC) as prospective regulatory nodes of neuronal excitability through modulation of the FGF14:Nav1.6 complex. Ingenuity Pathway Analysis shows convergence of these pathways on glycogen synthase kinase 3 (GSK3) and functional assays demonstrate that inhibition of GSK3 impairs excitability of hippocampal neurons. This combined approach provides a versatile toolkit for rapidly surveying PPI signaling, allowing the discovery of new modular pathways centered on GSK3 that might be the basis for functional alterations between the normal and diseased brain.

## Introduction

Kinases play fundamental cellular roles by serving as a nexus of enzymatic cascades governing intracellular protein signaling and genetic programs throughout the entire lifespan of the cell. Links between human diseases and dysfunction in kinase networks are numerous and multifactorial. In light of these connections, several kinase inhibitors have been evaluated as potential treatments for neurologic and psychiatric disorders.

Within the cell, kinases are critical for metabolism, intracellular signaling, transport, secretion, and many other vital cellular processes. Therefore, there is growing interest in targeting kinases through small-molecule inhibitors as a therapeutic strategy for brain disorders. Kinase inhibitors have been investigated as potential new therapeutics in Parkinson’s [1] and Alzheimer’s disease [2], and lithium, one of the first psychotropic drugs identified to be effective against bipolar disorder [3], is a potent inhibitor of glycogen synthase kinase 3 (GSK3), a multifunctional kinase implicated in schizophrenia, bipolar disorder, and depression [4–7]. Yet, despite clinical evidence suggesting that modulation of kinase pathways may affect therapeutic outcomes of brain disorders, the molecular targets of kinase pathways, especially in the CNS, remain poorly understood, limiting the understanding of disease causation and restricting development of new therapeutic strategies. Thus, there is an urgent need to rapidly survey kinase pathways to identify their relevant molecular targets that might be used as biomarkers of the disease state or as a base for therapeutic development.

The pore-forming alpha (α) subunit of the neuronal Nav channel is the key substrate of axonal and dendritic excitability within rapidly adapting brain networks [8,9]. The integrity and diversity of neuronal firing, synaptic transmission and activity-dependent remodeling of brain circuits is largely determined by the expression levels, sub-cellular localization, biophysical properties and post-translational modifications of the Nav channel [10,11] and its macromolecular complex of accessory and regulatory proteins. The functional specificity of these PPI and their post-translationally modified derivatives offer an asset for precise molecular interventions to restore maladaptive plasticity and aberrant firing in brain disorders [12,13].

Compelling evidence underlines the critical role of FGF14, a multivalent accessory protein of the Nav channel, in animal models and humans. Through direct monomeric binding to the Nav channel C-terminal tail, FGF14 forms a complex with the channel that is required for proper gating, expression and trafficking of the Nav channel to the axonal initial segment and consequently for neuronal excitability [14–20]. In humans, the naturally occurring FGF14^F145S^ mutation results in spinocerebellar ataxia 27 (SCA27), a severe motor and cognitive neurodegenerative disorder [15,21,22], and SNPs in the FGF14 gene have been associated with depression and schizophrenia [23,24]. Given the relevance of FGF14 for brain pathology, predicting and validating phosphorylation sites on FGF14 and the Nav channel, as well as elucidating the role of these post-translational modifications in the regulation of excitability, are essential steps toward discovering novel mechanisms at the base of brain disorders.

In recent studies, we reconstituted the FGF14 and Nav1.6 channel complex in live cells using the split-luciferase complementation assay (LCA) which allows surveying PPI using real-time light production as a relative binding read-out. As a result of a high throughput screening (HTS) of kinase inhibitors, we identified several GSK3 inhibitors as hits, and show that inhibition of GSK3 induces dissociation as well as subcellular redistribution of the native FGF14-Nav channel complex in hippocampal neurons [25]. Building on these results, we applied a combination of LCA and bioinformatics tools to evaluate 12 additional hits from the original HTS. Through a battery of dose-response studies of chemically-diverse inhibitors we also identified other kinase pathways modulating the FGF14:Nav1.6 channel complex assembly. Through bioinformatics, we found convergence of these additional kinases on the GSK3-pathway and show that GSK3 inhibitors suppress neuronal excitability in hippocampal neurons. The combination of a rapid bioluminescence-based assay for live cell studies and bioinformatics presented here provides a powerful toolkit enabling the discovery of new signaling pathways relevant for complex brain disorders. Furthermore, these results provide evidence for a novel signalosome that might control excitability through specific PPI placing the functional role of FGF14 in an even more complex physiological framework.

## Materials and Methods

**DNA Constructs.** All plasmids used in this study were previously described [25,26].

**Kinase inhibitors**. Inhibitors were purchased from chemical supply vendors (listed in **S1 Table**), weighed, and dissolved in DMSO to make freezable 20 mM stock solutions. The full description of vendors and stock and final concentration are listed in **S1 Table**.

**Cell culture and transient transfections.** HEK293 cells were maintained in DMEM (Invitrogen, Carlsbad, CA), supplemented with 10% fetal bovine serum, 100 U/ml penicillin, 100 μg/ml streptomycin, and 500 μg/ml G418 (Invitrogen), and incubated at 37°C with 5% CO2. Cells were transfected at 90–100% confluency using Lipofectamine 2000 (Invitrogen), according to manufacturer’s instructions. Prior to treatment with compounds, cells were maintained in serum-free, phenol-red free DMEM/F12 media.

**Primary neuronal cultures.** Banker’s style hippocampal neuron cultures were prepared from embryonic day 18 (E18) rat embryos using previously described methods [25,26]. Briefly, following trituration through a Pasteur pipette, neurons were plated at low density (1–5×10^5^ cells/dish) on poly-L-lysine-coated coverslips in 60 mm culture dishes in MEM supplemented with 10% horse serum. After 2–4 h, coverslips (containing neurons) were inverted and placed over a glial feeder layer in serum-free MEM with 0.1% ovalbumin and 1 mM pyruvate (N2.1 media; Invitrogen, Carlsbad, CA) separated by ∼ 1 mm wax dot spacers. To prevent the overgrowth of the glia, cultures were treated with cytosine arabinoside (5 μM; Calbiochem, La Jolla, CA) at day 3 *in vitro* (DIV) and used for patch-clamp electrophysiology at DIV 12–15.

**Bioluminescence Assays.** Cells (∼4.5 x 10^5^ per 24-well plates-mm dish) were transiently cotransfected with pairs of plasmids or single plasmids as indicated by using Lipofectamine 2000 (Invitrogen) according to the manufacturer’s directions. For bioluminescence assays, transfected cells (200 uL of medium) were transferred to 96-well white-walled plates 24 h prior to luminescence readings, 48 h after transfection. Prior to treatment with compounds, cells were incubated 30 min in serum-free, phenol-red free DMEM/F12 media. Subsequently, growth media were replaced with media containing appropriate drugs or vehicle and cells incubated for the times indicated. Luminescence readings were performed with the Synergy H4 Hybrid Multi-Mode Microplate Reader initiated by automated injection of 100 uL of substrate (in a 1:1 volume ratio) containing 1.5mg/mL of D-luciferin (final concentration = 0.75 mg/mL), followed by 3s of mild plate shaking, and measurements taken at 2 minute intervals with 0.5 s integration time for a total duration of 30 min. Raw signal intensity was computed from the mean value of peak luminescence and two adjacent time points for the biological replicates for a particular dose-compound dataset. Normalized signal intensity was expressed as percentage of mean signal intensity relative to control treated with 0.5% DMSO. Independent untreated controls, controls treated with vehicle, and Cluc-FGF14 mono-transfected HEK293 cells were performed for each 96-well plate for technical verification and to obtain positive and negative controls.

**Western blotting.** Transfected HEK293 (or HEK293-Nav1.6) cells treated for 1 h at 37°C with kinase inhibitors (or DMSO) were washed with phosphate-buffered saline (PBS) and buffer containing (in mM): 20 Tris-HCl, 150 NaCl, 1% NP-40. Protease inhibitor cocktail (set 3, Calbiochem) was added immediately before cell lysis. Cell extracts were collected, and sonicated for 20 sec then centrifuged at 4°C, 15,000 × g for 15 min, adding 4× sample buffer containing 50mM tris(2-carboxyethyl)phosphine (TCEP). Mixtures were heated for 10 min at 65°C and resolved on 4–15% polyacrylamide gels (BioRad, Hercules, CA). Resolved proteins were transferred to PVDF membranes (Millipore, Bedford, MA) for 1.5–2 h at 4°C, 75 V and blocked in TBS with 3% nonfat dry milk and 0.1% Tween-20. Membranes were then incubated in blocking buffer containing mouse monoclonal anti-Luciferase mAb (1:1000, Sigma-Aldrich, St. Louis, MO), mouse monoclonal anti-myc (1:1000; 9E10 clone Santa Cruz Biotechnology, Santa Cruz, CA), mouse monoclonal anti-PanNav channel (1:1000; Sigma Aldrich, St. Louis, MO), or rabbit polyclonal anti-calnexin (1:4000; Cell Signaling Technology, Danvers, MA) antibody overnight. Washed membranes were incubated with goat anti-mouse or goat anti-rabbit HRP antibody (1:4000–8,000; Thermo Scientific, Rockford, IL) and detected with ECL Advance Western Blotting Detection kit (GE Healthcare, Piscataway, NJ); protein bands were visualized using FluorChem HD2 System and analyzed with AlphaView 3.1 software (ProteinSimple, Santa Clara, CA).

**Immunoprecipitations.** Immunoprecipitations from HEK293-Nav1.6 cells were as previously described [25]. Cells were washed twice with PBS and lysed in the following lysis buffer: 20 mM Tris-HCl, 150 mM NaCl, and 1% NP-40 or Triton X-100. Protease inhibitor mixture (Cocktail #3; Calbiochem) was added immediately before cell lysis. Cell extracts were collected and sonicated for 20 s and centrifuged at 4°C, at 15,000 × *g* for 15 min. Supernatants were collected and incubated with rabbit anti-myc agarose beads (Sigma Aldrich) for 2 h at 4°C with agitation. After washing five times with lysis buffer, 2× sample buffer (Bio-Rad, Hercules, CA) containing 50 mM TCEP (tris(2-carboxyethyl) phosphine) was added. Lysates were then heated for 10–15 min at 65°C and resolved on 7.5% or 4–15% polyacrylamide gradient gels (Bio-Rad). Resolved proteins were transferred to polyvinylidene difluoride membranes (Millipore, Bedford, MA) for 2 h at 4°C and blocked in Tris-buffered saline with 5% skim milk and 0.1% Tween 20. Membranes were then incubated in blocking buffer containing a monoclonal anti-myc (1:1000; Santa Cruz Biotechnology, Santa Cruz, CA) or anti-PanNav channel (1:1000; Sigma) antibody overnight at 4°C. Washed membranes were incubated with goat anti-mouse HRP (1:5000–10,000) detected with ECL Advance Western Blotting Detection kit (GE Healthcare, Piscataway, NJ). Protein bands were visualized using FluorChem HD2 System and analyzed with AlphaView 3.1 software (ProteinSimple, Santa Clara, CA).

**Patch-clamp electrophysiology.** Whole-cell patch-clamp recordings were obtained from cultured rat hippocampal neurons at 12–15 DIV at room temperature (20–22°C) using a MultiClamp 700B amplifier (Molecular Devices), low-pass filtered at 2.2 kHz, and sampled at 20 kHz using a Digidata 1322A analog-to-digital interface and pClamp9 acquisition software (Molecular Devices). The extracellular bath solution contained (in mM) 140 NaCl, 4 KCl, 2 MgCl2, 2 CaCl2, 20 HEPES, and 10 glucose, pH 7.4; bicuculline (10 μM), NBQX (20 μM), and APV (100 μM) were added to block synaptic activity mediated by GABA, AMPA, and NMDA receptors, respectively. Recording pipettes (3–4 MΩ) were fabricated from borosilicate glass (WPI) using a two-step vertical puller PC-10 (Narishige), and filled with intracellular solution containing the following (in mM): 120 CH3KO3S, 10 KCl, 10 HEPES, 10 glucose, 2 MgCl2, 0.5 EGTA, 2 MgATP, and 0.5 Na3GTP, osmolarity 280–290, pH 7.3, adjusted with KOH. Seal formation and membrane rupture were done in voltage clamp mode at holding potential of -70 mV. After break-in cells were maintained at -70 mV holding potential in voltage clamp mode for ∼1 minute and then switched to current clamp mode. To acquire single action potential and passive properties all cells then were set to the membrane potential of -60 mV with injection of holding current. Neuronal single action potential was induced with a series of square current steps of 2.5 msec duration and increment of 20 pA. The action potential threshold was defined as the voltage at which the first time derivative of the rising phase of the action potential exceeds 10 mV/ms. The half-width was defined as the duration of single action potential measured at 50% repolarization. Passive membrane properties such as input resistance (R_in_) were measured with current-clamp recordings from a membrane potential of −60 mV. For determination of R_in_ the steady-state values of the voltage responses to a series of current steps from −120 to +20 pA with 20 pA increment per step and duration of 200 ms were plotted as a voltage–current relationship. R_in_ was calculated as the slope of the data points fitted with linear regression.

**Statistics.** Statistical values are given as mean and standard error of mean (mean ± SEM) unless otherwise stated. Positive controls (Cluc-FGF14-b and CD4-Nav1.6-Nluc) and negative controls (Cluc-FGF14-b) treated with 0.5% DMSO were included for each treatment group; positive untreated controls were also evaluated for technical verification. Statistical significance among treated vs. control groups (p<0.05) was assessed by Student’s *t*-test (equal-variance) using GraphPad Prism 6 (GraphPad Software, La Jolla, CA).

**Dose-response analysis.** Dose-response curves were obtained by fitting the data with a nonlinear regression:

$$A+\frac{B-A}{1+10^{\log(x_{0}-x)}H}$$ [Equ 1]

where x is log_10_ of the compound concentration in M, x_o_ is the inflection point (EC_50_ or IC_50_), A is the bottom plateau effect, B is the top plateau effect, and H is the Hill slope. Dose-response data were normalized by dividing all intensities by the average peak intensity observed for the entire dose-response range, independently for each compound. Inhibitors that increased FGF14:Nav1.6 interaction with increasing doses were classified as agonists; inhibitors that decreased FGF14:Nav1.6 interaction were classified as inverse agonists. Compound efficacy was computed by:

$$Efficacy=\frac{1-A}{A}$$ [Equ 2]

where A is the bottom plateau effect, as derived through nonlinear regression of [Equation 1]. Inverse agonists with an efficacy value greater than 1 were classified as full inverse agonists; those that fail to meet this criterion were classified as partial inverse agonists. All graphs were generated with Graphpad Prism 6, and subsequently processed for visual presentation with Adobe Illustrator CS6.

**Bioinformatics.** Dose-response heat maps and hierarchical clustering was performed with R 3.1.0 using the heatmap() and hclust() functions, respectively. (Functions in R are denoted by the notation “function()”, where the appropriate parameters, including datasets, configuration options, and visual layout for the analysis are input between the parentheses). Clusters were generated using the *complete linkage* method, generating a dendrogram where the distance between two clusters was defined as the maximum distance between their individual components (differences in normalized interaction strength between Nav and FGF14 for each dose-response assessed). The contributions of each dose-response category were equally weighed. Principal component analysis of dose-response data was performed with R 3.1.0 using the princomp() function and visualized with internal graphing tools. Principal components were computed using the eigen() function on the covariance matrix, and the three most significant components visualized. Inhibitors and kinases were visualized with a Scree plot, with lines denoting individual inhibitors or kinases and the contributions of each principal component, and spheres denoting the aggregate response for a particular dosage. For Ingenuity Pathway Analysis (IPA), the list of major kinase targets of each inhibitor (**S1 Table**, leftmost column) was input as a gene list in the IPA interface, and the closest match selected in the IPA database. The “Connect” tool in Path Designer was used to generate an interaction network, showing either only direct interactions, or direct and indirect interactions. The interaction network was subsequently submitted to the Pathway Analysis engine, with the default options selected to detect both direct and indirect interactions across all species in the database. Generated raw heatmaps, dendrograms, Scree plots, and interaction networks were subsequently processed for visual presentation with Adobe Illustrator CS6.

## Results

**Fig 1:**
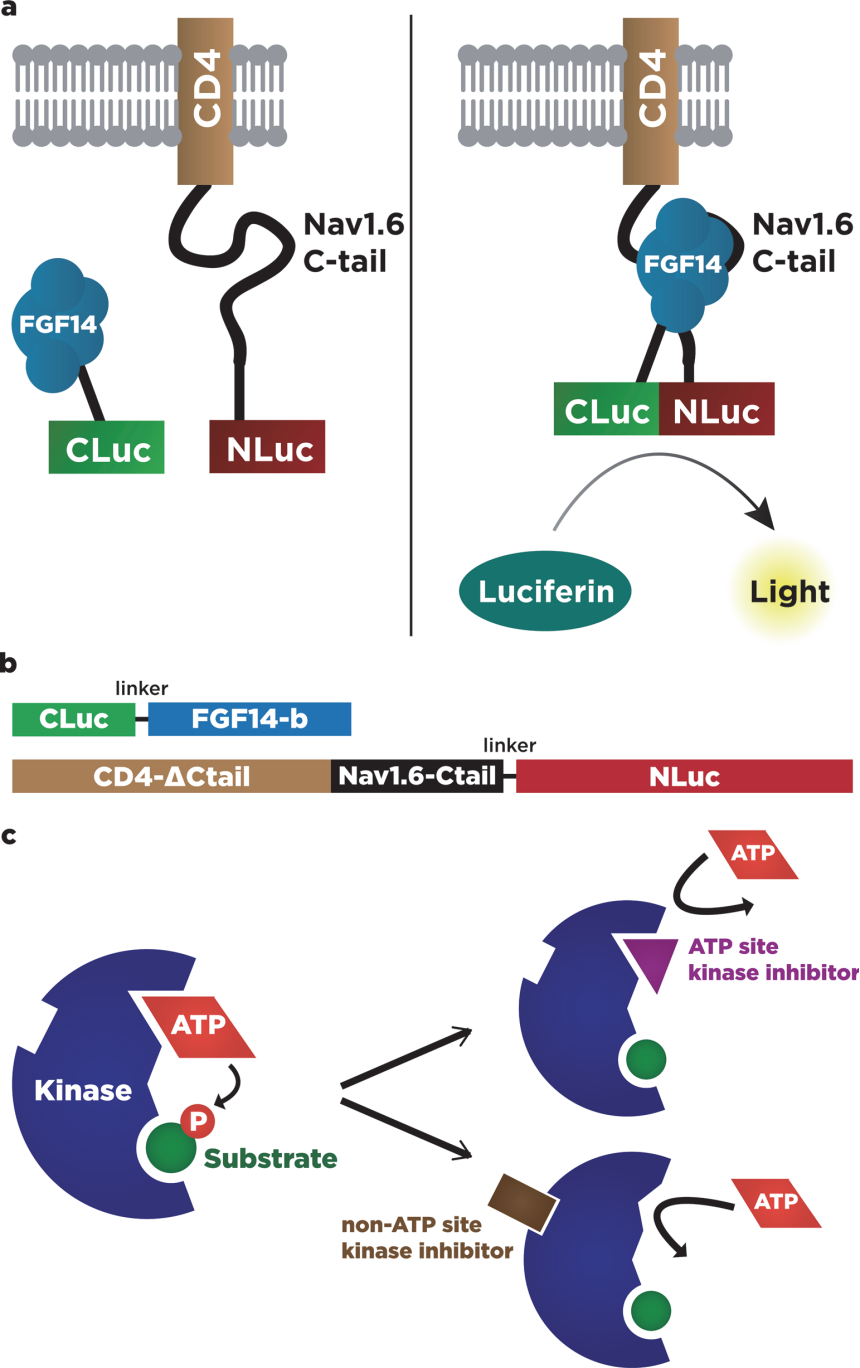
Using LCA to measure real-time interaction between FGF14 and Nav1.6 C-tail in live cells. (**A**) Using the bioluminescence-based luciferase complementation assay (LCA) to measure protein:protein interactions. Two proteins of interest (FGF14, and CD4-Nav1.6-Ctail in this example) are fused to Cluc and Nluc fragments of *Photinus* luciferase. Upon interaction of the protein components, Nluc and Cluc fragments reconstitute into functional luciferase enzyme, which produces luminescence in the presence of luciferin substrate. The intensity of luminescence is linear to the strength of the protein:protein interaction identified. **(B)** Schematic of constructs used for LCA experiments, to scale. **Top**: Cluc (AAs 398–550), linker (GGGSSGGGQISYASRG), FGF14-b (AAs 1–252). **Bottom:** CD4-ΔCtail (AAs 1–395), Nav1.6-Ctail (AAs 1763–1976), linker (QISYASRGGGSSGGG), Nluc (AAs 2–416). **(C)** Schematic of protein inhibition by kinase inhibitors. ATP-competitive kinase inhibitors block the ATP-binding site of the target kinase, preventing the transfer of phosphate groups to the substrate. Non-ATP competitive kinase inhibitors work through other mechanisms, such as changing the conformation of the ATP-binding site to prevent docking of ATP.

**Fig 2:**
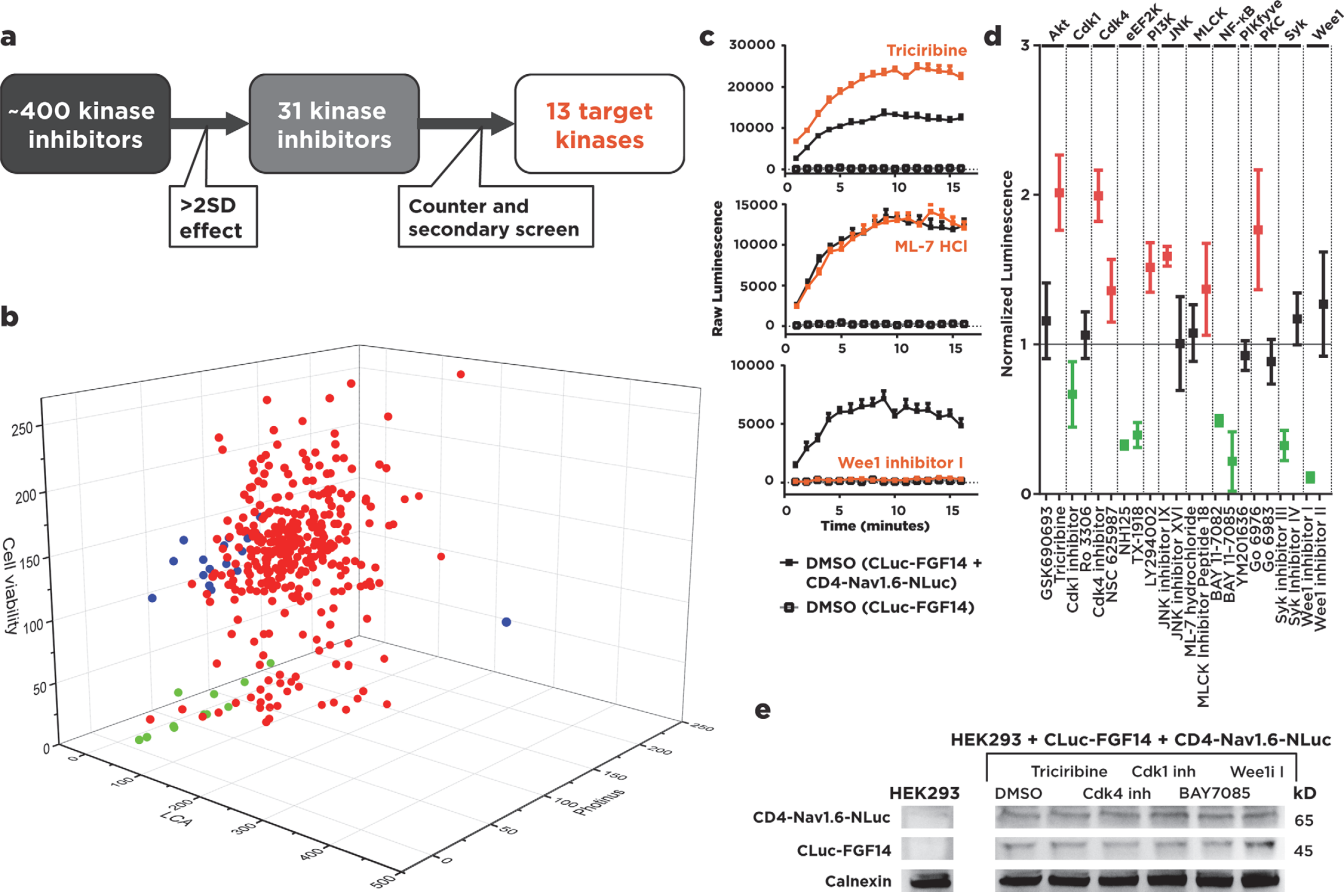
Split luciferase assay-based screen identifies new phospho-regulatory pathways upstream of the FGF14:Nav1.6 channel complex. **(A)** Schematic diagram of the large-scale kinase inhibitor libraries-based screen detected by bioluminescence. Counter and secondary screenings used include validation of cell viability, measurement of inhibitor activity against intact *Photinus* luciferase, and siRNA validation of target kinases. **(B)** 3D plot of screening results by split-luciferase signal (LCA), *Photinus* luciferase assay, and cell viability. Results that passed screening are shown in blue; results that passed screening but were rejected based on counter and secondary screens are shown in green. **(C)** Example time series data for increased (triciribine), unchanged (ML-7 Hydrochloride) or decreased (Wee1 inhibitor) luminescence relative to control (Cluc-FGF14 / CD4-Nav1.6-Nluc) shown, at a final concentration of 50 μM. **(D)** Results of the screen are represented as peak areas (±1 time point of peak) normalized to % luminescence values of DMSO treated controls (FGF14:Nav1.6 complex) at a final concentration of 50 μM. Data shown is mean ± 95% CI. Significant increase (red) or decrease (green) of luminescence, relative to the control signal (p < 0.05) shown. **(E)** Western blot analysis of HEK293 and transfected HEK293 cells, visualized with anti-calnexin (Calnexin) and anti-luciferase (CD4-Nav1.6-Nluc and Cluc-FGF14) antibodies. Transfected HEK293 cells were treated with 0.5% DMSO or inhibitors dissolved in 0.5% DMSO at an empirically determined concentration to minimize effects on cell viability (calnexin). Use concentrations are as follows: Triciribine (25 uM), Cdk4 inhibitor (15 uM), Cdk1 inhibitor (25 uM), BAY 11–7085 (25 uM), Wee1 inhibitor (15 uM).

Protein-fragment based complementation assays have recently emerged as a powerful, versatile toolkit for the exploration of PPI between expressible proteins [26,27]. The bioluminescence-based luciferase complementation assay (LCA) **(Fig. 1)** relies on the complementation of protein fragments linked to N-terminal and C-terminal fragments of firefly luciferase respectively, and produces a luminescence readout that is robust, reversible, has a high signal-to-noise ratio, and is amenable to high-throughput scaling. These factors make it an ideal system to detect PPI, protein localization, intracellular protein dynamics, and protein activity in living cells and animals [28]. In this study, we used LCA to reconstitute the FGF14 and Nav1.6-C-tail complex (**Fig. 1A**) upon transient transfection of CLuc-FGF14 and CD4-Nav1.6-C-tail plasmids (**Fig. 1B**) into HEK293 cells and further characterized the activity of structurally-diverse kinase inhibitors (**Fig. 1C**) identified previously through a high-throughput screening (HTS). In the original screening, 31/384 compounds were declared hits based on statistical criteria accounting for magnitude and reproducibility of the effect (up or down-regulation of the FGF14:Nav1.6 complex) and on lack of interference with the luciferase reporter activity and of cell toxicity **(Fig. 2A-2B)** [25].

By applying these screening criteria to the observed hits, we identified 13 kinase pathways corresponding to the primary kinase target(s) modulated by screened compounds (**Fig. 2A-2B**). In addition to GSK3 that was pursued in the original study, we identified the following pathways: Akt, Cdk1, Cdk4, eEF2K, PI3K, JNK, MLCK, NF-kB, PIKfyve, PKC, Syk and Wee1 (**Fig. 2D**).

To provide a more accurate profile of the identified targets, we consulted literature references and searched chemical suppliers to identify two selective inhibitors that had been previously validated against the desired kinase pathway. We ordered the respective compounds (EMD Millipore, Selleckchem, Tocris, Santa Cruz Biotech, Sigma-Aldrich, Cayman Chemicals), and created 20 mM working stock solutions in DMSO (**Fig. 2A**, **S1 Table**). Compounds were first evaluated at a single final working concentration of 50 μM (in 0.5% DMSO) applied for 1–2 hours prior to the LCA to HEK293 cells previously transfected with CLuc-FGF14 and CD4-Nav1.6-NLuc constructs. Three representative examples of real-time LCA responses are shown in **Fig. 2C** with the Akt inhibitor, triciribine, increasing FGF14:Nav1.6 complementation (**top panel**), ML-7 hydrochloride, a target of MLCK, producing marginal effects on the complex (**middle panel**) and Wee1 inhibitor, a potent inhibitor of Wee1 kinase, greatly suppressing the FGF14:Nav1.6 complex formation (**bottom panel**). At a working concentration of 50 μM, we successfully identified distinct clusters of compounds that up-regulated and down-regulated the FGF14:Nav1.6 interaction (**Fig. 2C-2D**); 7 of the compounds identified (Triciribine, Cdk4 inhibitor, NSC 625987, LY294002, JNK inhibitor IX, MLCK peptide 18, Gö 6976) significantly up-regulated the FGF14:Nav1.6 complex, while 7 compounds (Cdk1 inhibitor, NH125, TX-1918, BAY 11–7082, BAY 11–7085, Syk inhibitor III, and Wee1 inhibitor I) significantly disrupted the FGF14:Nav1.6 interaction. Interestingly, the activity of compounds targeting the same primary kinase was variable, with certain kinases, such as PKC, displaying dramatically opposing patterns of regulation or even exhibiting a lack of significant effect (GSK690693, Ro 3306, JNK inhibitor XVI, ML-7 hydrochloride, YM201636, Gö 6983, Syk inhibitor IV, and Wee1 inhibitor II) depending on the specific compound used. This necessitated a closer look at the actual dose-response profile of the compounds under investigation. Importantly, these effects were not explained by alterations in expression levels of CLuc-FGF14 and CD4-Nav1.6-Nluc of inhibitor-treated cells at physiologically relevant concentrations (**Fig. 2E**).

**Fig 3:**
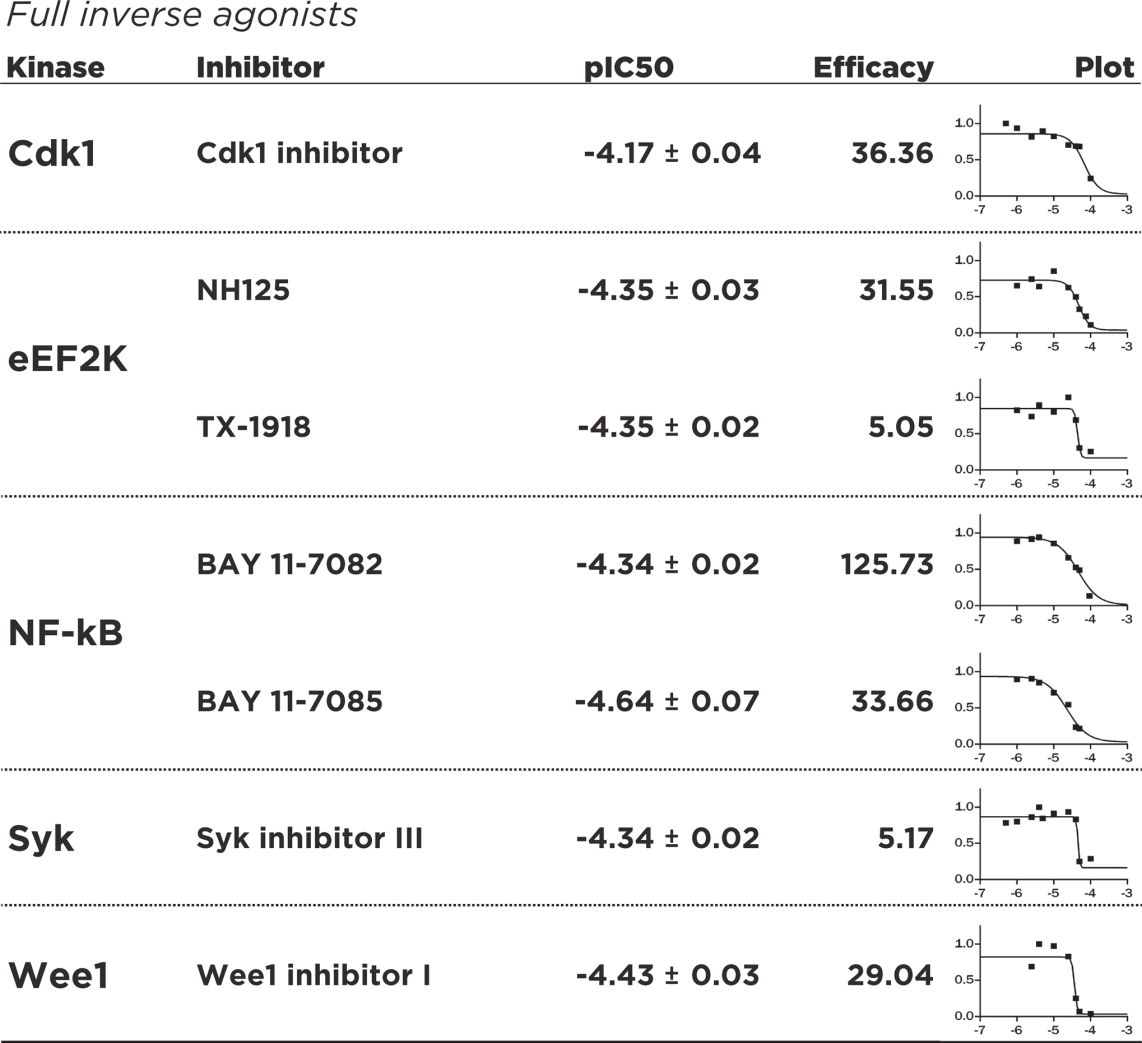
Dose-response studies of identified full inverse agonists of FGF14:Nav1.6 regulatory pathways. Fitting was performed with nonlinear regression using Graphpad Prism 6 (**Statistics**). Indicated pIC50 and pEC50 were derived from best fit nonlinear regression after a maximum of 1000 iterations. Full inverse agonists were defined as compounds that act as inverse agonists (inhibit FGF14:Nav1.6 complementation with increasing dose) and have an efficacy value of greater than 1. **Plot, X-axis:** log_10_([Inhibitor]), **Y-axis:** Percent of intensity, normalized to peak observed raw intensity for each compound.

**Fig 4:**
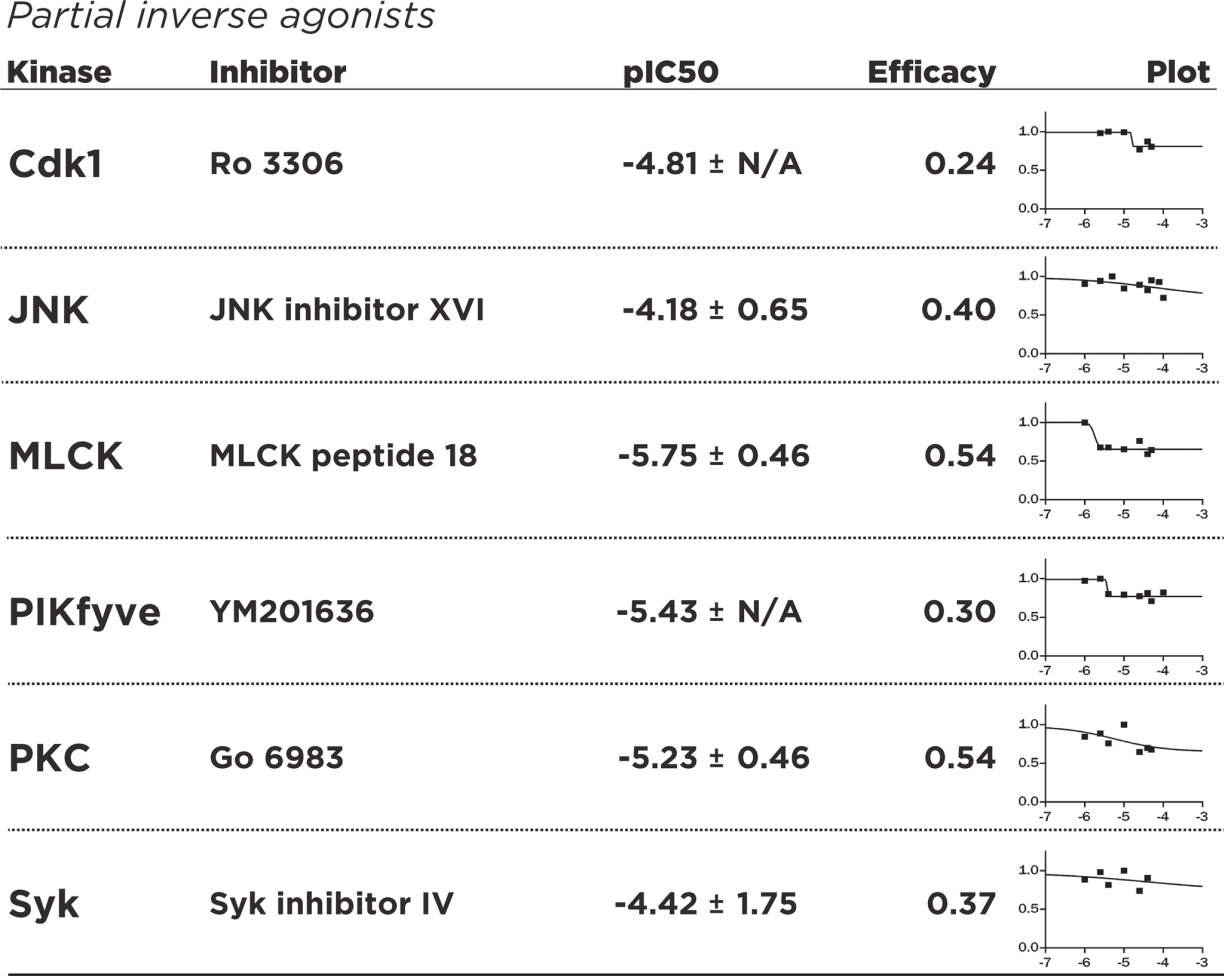
Dose-response studies of identified partial inverse agonists of FGF14:Nav1.6 regulatory pathways. Fitting, pIC50/EC50 calculation, axes as in Fig. 3. Partial inverse agonists were defined as compounds that act as inverse agonists (inhibit FGF14:Nav1.6 complementation with increasing dose) and have an efficacy value of less than 1.

**Fig 5:**
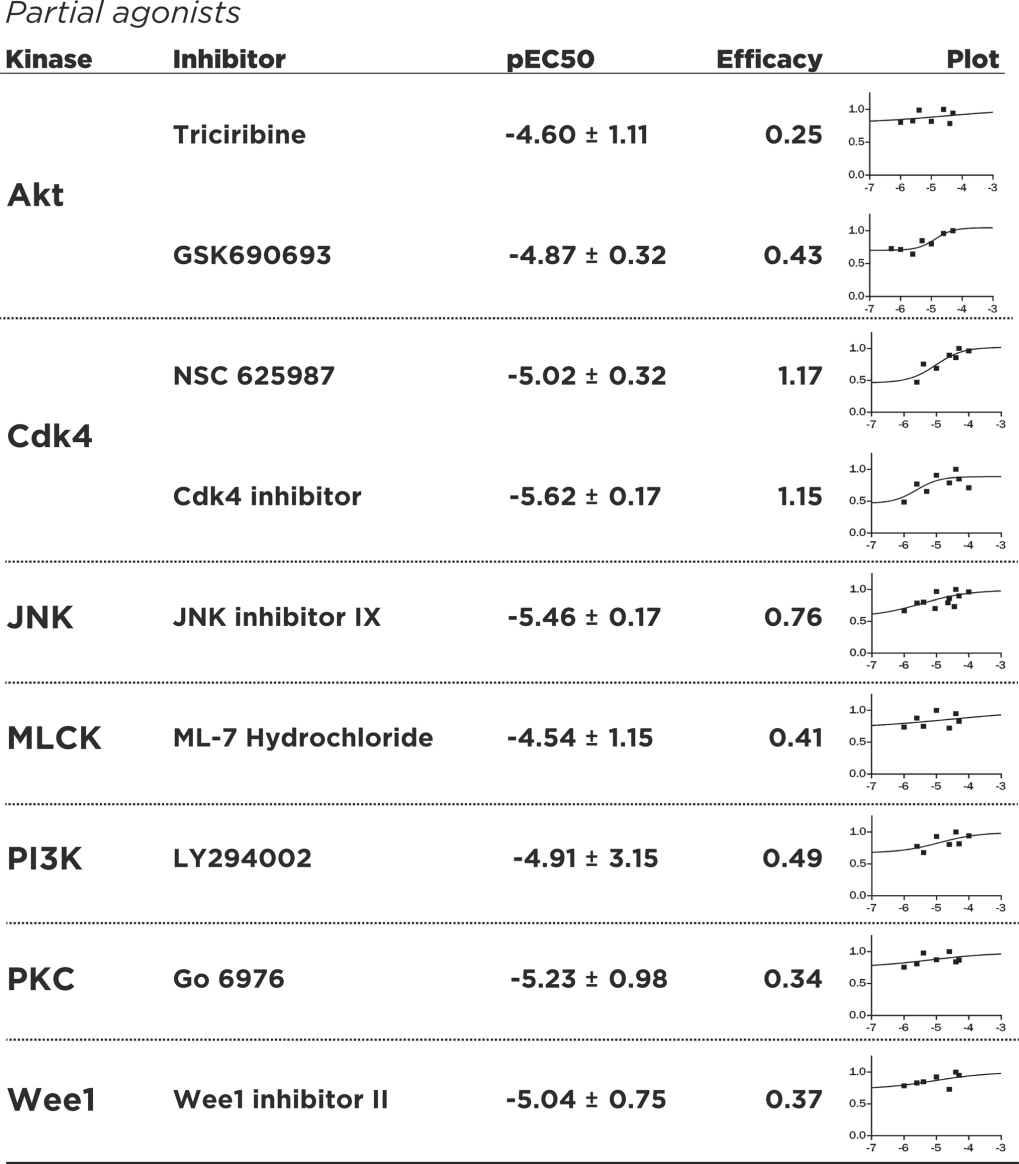
Dose-response studies of identified partial agonists of FGF14:Nav1.6 regulatory pathways. Fitting, pIC50/EC50 calculation, axes as in Fig. 3. Partial agonists were defined as compounds that act as agonists (promote FGF14:Nav1.6 complementation with increasing dose).

Investigating the dose-response characteristics of inhibitors can reveal novel insights into pharmacological mechanisms. Recent investigations into the clinical possibilities of kinase inhibitors and continuing efforts towards drug discovery of better inhibitors underlines modern thinking of kinase inhibitors as both agonists and antagonists of complex cellular pathways [29]. Towards this aim, we have adapted the concept of efficacy, a pharmacological parameter quantifying receptor occupancy by drug molecules [30], as a quantitative way to determine the ability of kinase inhibitors to target the FGF14:Nav complex at various dose concentrations. Additionally, we define an “agonist” of the FGF14:Nav system as a compound that increases the interaction of FGF14 with Nav as a function of increasing dosage, and an “inverse agonist” as a compound that similarly decreases the FGF14:Nav interaction. Dose-dependent experiments were performed with n = 6 biological replicates for up to 7 working concentrations (50 uM, 40 uM, 25 uM, 10 uM, 4 uM, 2.5 uM, 1 uM), and runs for all experiments were combined to produce a dose-response curve for each inhibitor. The dose-response data were subsequently normalized with respect to the maximum peak intensity observed for each compound to determine the degree of agonism/inverse agonism. The dose-response data hint at the heterogeneity of kinase inhibition, and the need to establish effective, working concentrations for further study. The inhibitors Cdk1 inhibitor, NH125, TX-1918, BAY 11–7082 and BAY 11–7085, Syk inhibitor III, and Wee1 inhibitor I produced a dose-response consistent with pharmacological full inverse agonists, with FGF14:Nav interaction almost absent at saturating concentrations and high (e>1) efficacy values **(Fig. 3)**, making them most suitable for studying the dose-dependent inhibition of the FGF14:Nav complex. Conversely, inhibitors such as Ro 3306 and Go 6983 can be classified as partial inverse agonists, with substantial residual FGF14:Nav response at saturating concentrations and low (e<1) efficacy **(Fig. 4)**. This is probably explained through activation of competing or compensatory pathways that may increase FGF14:Nav binding, while other inhibitors such as triciribine and NSC 625987 produce responses consistent with partial agonism of the FGF14:Nav1.6 complex, useful in studying the phenotype of increased FGF14:Nav binding **(Fig. 5)**. In addition, the observed effective IC50 values for each of the inhibitors differs markedly from *in vitro* reported values (**S1 Table**), many of which are in the low nanomolar range. These results demonstrate the hazard of relying on *in vitro* IC50 values to gauge effectiveness in actual cells and tissues, as well as the need to conduct dose-response screenings independent of manufacturer-provided data for a particular biological system of choice.

**Fig 6:**
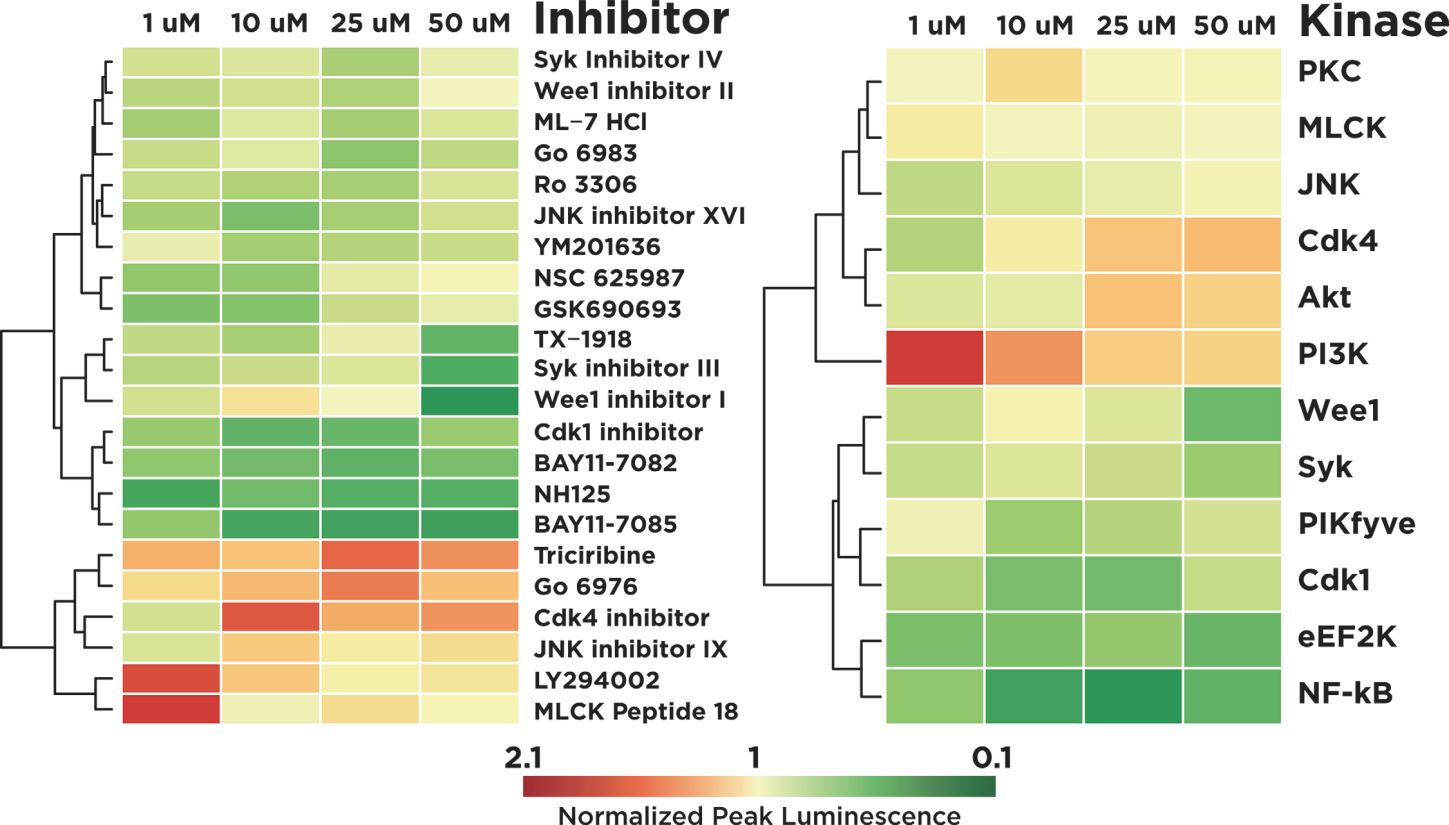
Hierarchical clustering of inhibitors for FGF14:Nav1.6 regulatory pathways. **A)** Heatmap and hierarchical clustering for individual inhibitors. Red, increased intensity relative to DMSO control. Green, decreased intensity. **Left**, Hierarchical clustering, based on differences in normalized interaction strength between Nav1.6 and FGF14 for each inhibitor, with equal weighing of all categories. **B)** Heatmap and hierarchical clustering for primary kinase targets of each inhibitor, derived from geometric averaging of all inhibitors of each primary kinase.

Large-scale analysis of kinase inhibitors has yielded novel insights into the function and dysregulation of cellular pathways [31,32]. However, data about the functional Nav-centered interactome in neurons are lacking. Simultaneously, dose-response experiments generate large volumes of data, but can be difficult to meaningfully interpret. To attempt to address both of these problems, we constructed a heatmap of our dose-response data at four selected concentrations (50 uM, 25 uM, 10 uM, and 1 uM) representative of the dynamic range of our data, and applied hierarchical clustering methods to infer inhibitors that demonstrate similar dose-response patterns. Even through this unsupervised approach, several notable patterns emerged; most notably, hierarchical clustering of inhibitors grouped triciribine in close proximity with LY294002, as well as PI3K with PIKfyve kinase, corresponding to the well-studied Akt/PI3K/PIKfyve pathway which is known to negatively regulate GSK-3 (Fig. 6). The inhibitor Gö 6976 is highly associated with this pathway, suggesting that PKC may also be a major constituent in this pathway. JNK inhibitor IX and the Cdk4 inhibitor are also closely associated with PKC, forming a JNK/CDK4/PKC network that could be important for inflammosome formation and stress signaling [33,34]. Interestingly, the Wee1 inhibitor, a potent suppressor of Wee1 kinase, a serine-threonine kinase with important implications for neuronal polarity [35], is closely clustered with the Akt/PI3K pathway, suggesting important functional links for this key regulator of cell cycle progression.

**Fig 7:**
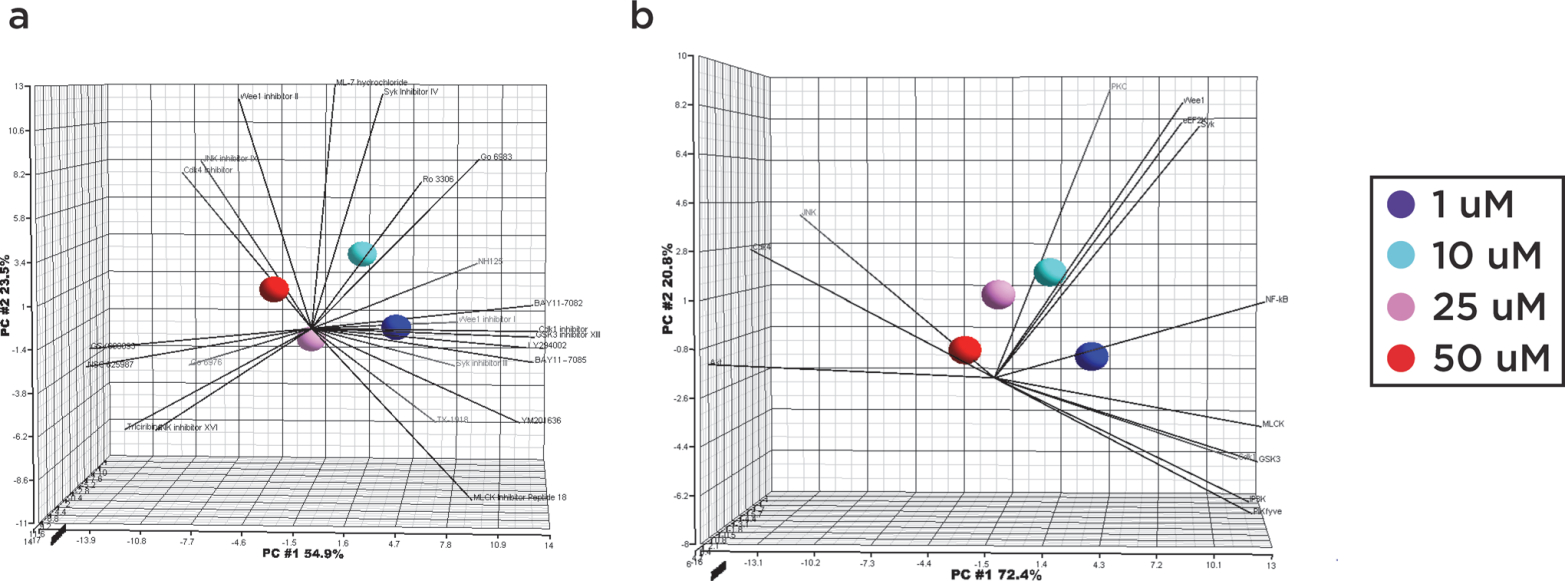
Principal component analysis of inhibitor/kinase dose response profiles. Computed principal components are visualized with a Scree plot, with the top 3 principal components (PC #1, PC #2, PC #3) denoted by orthogonal axes and line segments denoting individual inhibitors or kinases. The first principal component contributes the most (54.5% for inhibitors and 70.5% for kinases) and is denoted by the horizontal axis. Spheres denote the composite response of a single dose category (1, 5, 25, 50 uM). **A)** Response profile of inhibitors. **B)** Response profile of kinases.

**Fig 8:**
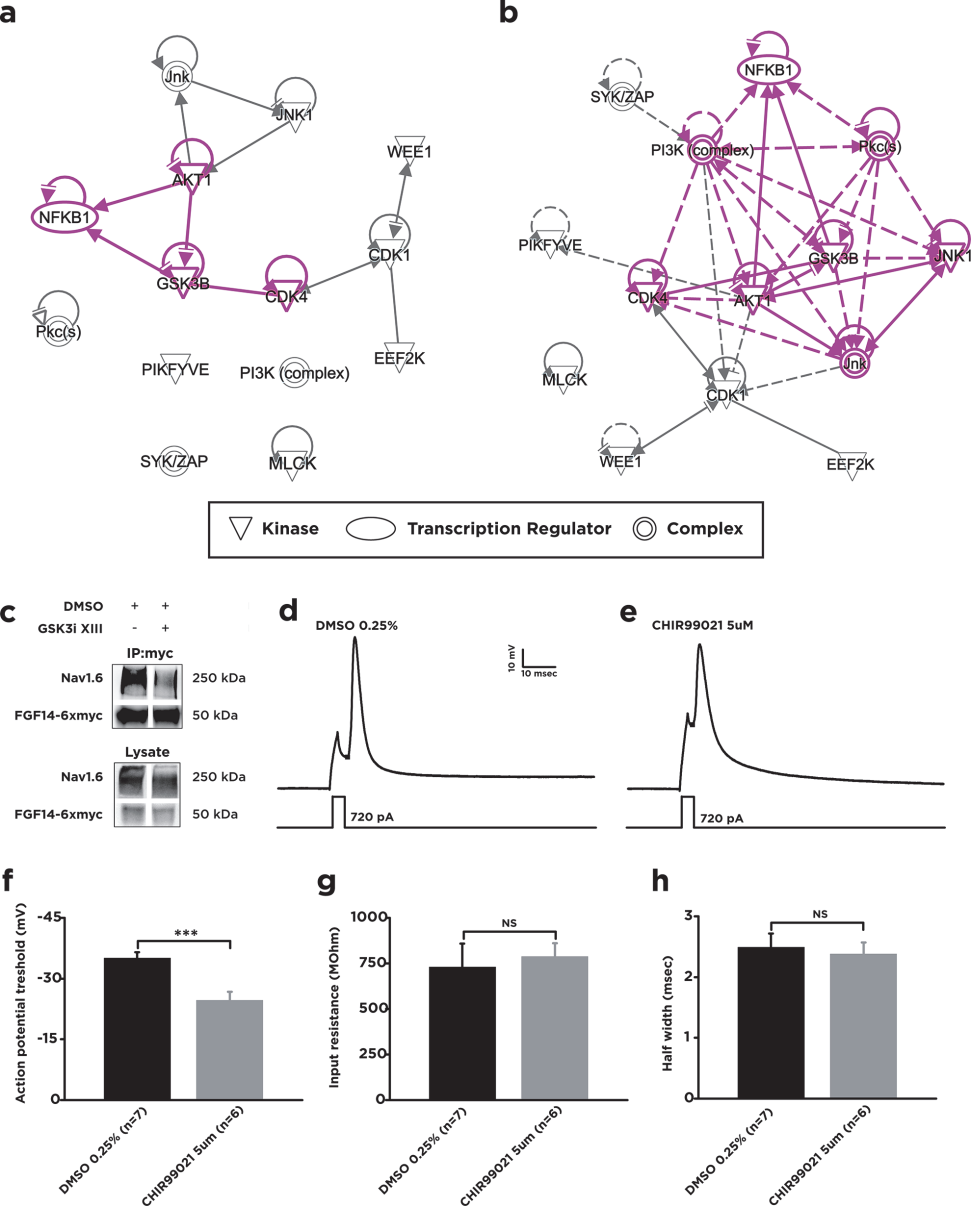
Bioinformatics analysis of a GSK-3 centered kinase regulatory network. For protein interaction networks, Ingenuity Pathway Analysis (IPA) was applied to the list of identified major kinase targets for all of the inhibitors tested, in addition to GSK-3, and an unbiased network was generated using the “Connect” algorithm and subsequently submitted to the pathway analysis engine. Both direct (solid) and indirect (dashed) interactions, as classified by Ingenuity, are shown; the sub-network with edge length of 1 to GSK-3 is additionally highlighted (purple). **A)** Interaction network, showing direct interactions only. **B)** Interaction network, showing both direct (solid) and indirect (dashed) interactions. **C)** Western blot analysis of co-immunoprecipitation (*IP*:*myc*) and cell lysate from HEK293-Nav1.6 cells transfected with FGF14–6×myc. GSK3 inhibitor XIII (25 μM) treatment reduces the co-immunoprecipitated fraction of Nav1.6 without affecting FGF14–6×myc. **D)** and **E)** representative traces showing effect of 12 hour treatment with ether DMSO 0.25% (**D**) or CHIR99021 5μM (**E**) on neuronal excitability in cultured hippocampal neurons DIV 12–15. Single action potentials were evoked by brief (2.5 ms) depolarizing current injections. Grey squares indicate action potential threshold. **F)** CHIR99021 increases action potential threshold in cultured hippocampal neurons. Results represent mean ± SEM. n = 7 (DMSO), n = 6 (CHIR990221). *p<0.05, Student *t*-test. **G)** and **H)** CHIR99021 has no effect on input resistance (**G**) and action potential half width (**H**) threshold in cultured hippocampal neurons. Results represent mean ± SEM. n = 7 (DMSO), n = 6 (CHIR990221), NS—non significant, p>0.05, Student *t*-test.

In the exploratory analysis of multidimensional datasets, bioinformatics tools such as principal component analysis (PCA) and protein interaction networks are useful to provide a higher-order understanding of the dataset at hand. Specifically, the application of PCA allows for the determination of which inhibitors are most closely associated with the observed effect at low, moderate, and high doses. Applying PCA to the dose-response data reveals that the response profiles of triciribine, JNK inhibitor XVI, Go 6976, NSC 625987, GSK690693, Cdk1 inhibitor, and JNK inhibitor IX are most closely associated with higher dosages (25 and 50 uM) (**Fig. 7A**), while the rest of the tested inhibitors, except for Wee1 inhibitor II, ML-7 hydrochloride and Syk Inhibitor IV, are closely associated with low dosages (1 and 10 uM). Similarly, the kinases most associated with high dosages include JNK, Akt, and Cdk4; at moderate doses, Wee1, eEF2K, and Syk, and at lower doses, NF-kB, MLCK, Cdk1, PIKfyve and IP3K (**Fig. 7B**). These results might suggest that the inhibitors and their targeting kinases that associate with lower doses may act through direct, unopposed biological mechanisms, while inhibitors and kinases that associate with higher doses may act through indirect mechanisms, or through mechanisms with compensatory behavior. When the kinase targets of these inhibitors were analyzed through Ingenuity Pathway Analysis (IPA), GSK3 emerged as a critical node in both the direct and indirect interaction networks generated (**Fig. 8A, 8B**), with the sub-network directly proximal to GSK3 encompassing over half of the total kinase list (**Fig. 8B**).

To provide further validation of the GSK3 centrality toward the FGF14:Nav1.6 complex outside the LCA platform, we applied co-immunoprecipitation. In HEK293-Nav1.6 cells treated with 25 uM GSK3 inhibitor XIII we found that the level of co-immunoprecipitation of full-length Nav1.6 and FGF14 is decreased compared to DMSO controls, corroborating the idea that inhibition of GSK3 adversely affects the assembly of the FGF14:Nav1.6 channel complex (**Fig. 8C**).

FGF14 is an integral component of the AIS in hippocampal neurons [25] where it forms a complex with native Nav channels and contributes to action potential generation [14–16]. Thus, we posited that GSK3 inhibitors in hippocampal neurons, where both FGF14 and Nav1.6 are abundantly expressed, might reduce the activity of Nav channels leading to suppressed intrinsic excitability. Accordingly, we found that hippocampal neurons treated with the GSK3 inhibitor, CHIR99021 (5 μM), exhibited a significant depolarizing shift (∼10 mV) in action potential threshold compared to DMSO-treated (0.25%) control neurons (-35.1±1.5 mV, n = 7 cells in the DMSO-treated group versus -24.7±2.1 mV, n = 6 cells in CHIR99021-treated group; p = 0.0015 with Student *t*-test, **Fig. 8D, E, F**). Other parameters, such as R_in_ and action potential half-width, considered as a reflection of Ca2+ and K+ channels activity, were unaffected (R_in_: 729.5±128.9 MΩ, n = 7 cells in DMSO-treated group versus 787.1±73.2 MΩ, n = 6 cells in CHIR99021-treated group; p = 0.71 with Student *t*-test, **Fig. 8G**; action potential half-width: 2.5±0.2 ms, n = 7 cells in DMSO-treated group versus 2.4±0.2 ms, n = 6 cells in CHIR99021-treated group; p = 0.72 with Student *t*-test, **Fig. 8H**). These observations suggest that inhibition of GSK3 affects neuronal excitability through shifting of the action potential threshold to a more positive value, likely through modulation of voltage-gated sodium channel function.

## Discussion

In this study we identified signaling pathway clusters including PI3K/Akt/GSK3, PKC and Wee1 converging on the FGF14:Nav1.6 channel complex. Small molecule inhibitors targeting these kinases exert effects of opposite direction and different magnitude on the FGF14:Nav1.6 complex assembly, suggesting that phosphorylation of either FGF14 and/or the C-terminal tail of Nav channels control the strength of PPI within this macromolecular complex. Previously, we have shown that FGF14, an intracellular fibroblast growth factor linked to neurodegenerative brain disorders [21] and altered synaptic transmission and plasticity [19], fundamentally controls channel gating and localization of Nav channels at the AIS, and is required for proper neuronal firing [14–16]. Kinase-dependent reversible phosphorylation of Nav channels has profound effects on action potential initiation, dendritic excitability, and other aspects of neuronal excitability [36]. PKA and PKC have been shown to phosphorylate several Ser residues on the interdomain I-II and III-IV linkers of Nav1.2, strongly reducing current and increasing firing thresholds [36–38]. Thus, through PPI-dependent regulation and direct effects on Nav channels, kinases might decode cellular signaling into a complex pattern of neuronal activity.

The study presented here stems from a HTS run against the FGF14:Nav1.6-C-tail channel complex. As a result of that study, we identified GSK3 as a target, showing that inhibition of GSK3 suppresses the relative binding strength of FGF14 to the Nav1.6 C-tail and destabilizes and disrupts cellular targeting of the FGF14:Nav complex to the AIS [25]. In the present study, we provide further verification of 12 additional kinases from the HTS and, through dose-response pharmacology, unsupervised network modeling and IPA, identified the PI3K/Akt pathway, the cell-cycle regulator Wee1 kinase and PKC as clusters (**Fig. 6** and **Fig. 7**) converging on GSK3 (**Fig. 8B**). Notably, we show that inhibition of Akt, a negative regulator of GSK3, increases FGF14:Nav1.6 complex assembly, and that LY294002, a small molecule inhibitor of PI3K, which activates Akt, also increases FGF14:Nav1.6 complex formation, supporting a PI3K/Akt/GSK3 directionally-mediated signaling cascade.

Similar to Akt inhibitors, the Wee1 inhibitor I leads to an increase in FGF14:Nav1.6 complementation at lower concentrations. Wee1 is a serine-threonine kinase that when dysfunctional results in the loss of neuronal polarity and is implicated in neurodegenerative changes found in Alzheimer’s [35,39]. Significantly, Wee1 is negatively regulated through ubiquitination by SCF^β-TrCP1/2^, an E3 ubiquitin ligase recruited by a complex containing GSK3 [40,41]. Furthermore, Wee1 is a strong inhibitor of cyclin-dependent kinases, which contribute to the repression of Rb protein, which may increase the activity of Akt [42].

Protein kinase C, a family of serine/threonine kinases of fifteen distinct isoforms, is highly enriched in the brain and other nervous tissues, and has crucial roles in ion channel modulation, neurotransmitter release, receptor modulation, synaptic potentiation and depression, and neuronal survival [43]. PKC is an important component of the pathophysiology of bipolar disorder, with overexpression in the brains and platelets of bipolar patients [44,45]. It is also correspondingly reduced in rats chronically treated with lithium, a commonly used mood stabilizer whose clinical efficacy is attributed to GSK3 inhibition [46]. Specific isotypes of PKC are known to be phosphorylated by PDK1 in a tightly regulated, PI3K-dependent manner [47], and this interaction is important for PI3Kγ-mediated oxidant signaling through NF-κB activation [48]. Furthermore, GSK-3β is selectively phosphorylated by PKC isoforms, resulting in its inactivation and corresponding upregulation of GSK-3β inhibited pathways [49]. Together, these mechanisms contribute to fine-tuned regulation of GSK3 activity accompanied by modulation of the FGF14:Nav1.6 complex.

GSK3 is a kinase whose dysfunction is central to depression, schizophrenia, and bipolar disorder [5,50–52], and selective GSK-3 inhibitors, largely ATP competitors, have been reported to have an antidepressant-like effect in mice [53,54] with modulation of the upstream PI3K-Akt-GSK3 pathway also playing a critical role [55,56]. S/T phosphorylation sites that match a GSK3 motif are found both on FGF14 and Nav1.6. The T^1966^ residue in the C-tail of Nav1.2 has been validated *in vivo* and it lies in a putative GSK3 phoshorylation motif, where the S or T target residues are spaced by two or three amino acids, preferentially prolines followed by a S/T residue (S/TpXXXS/T and S/TpXXS/T) [57,58]. This residue is conserved in Nav1.6 and might be a candidate for the effects of GSK3 inhibitors that we observed in this study. On the FGF14 side, T229 matches a putative GSK3 site, but no information is available on its phosphorylation status either *in vitro* or *in vivo*. In general, GSK3 phosphorylation sites that could influence the assembly of the FGF14:Nav1.6 complex could be a direct link to extracellular signaling through tyrosine-kinase receptors, G-protein coupled receptors or other signal transduction pathways upstream of the PI3K/Akt/GSK3 pathway. As such, they provide candidate targets for homeostatic regulation of firing and mechanistic links to brain circuitry hyperactivity found in brain disorders.

Although the most prominent effects on the FGF14:Nav1.6 channel complementation were found with the inhibitors of the PI3K/Akt/GSK3 pathway, Wee1 and PKC, significant activity was observed for other kinase inhibitors, including those targeting JNK, Cdk4, NF-κB, and eEF2K. A subfamily of the diverse MAP kinases, c-Jun N-terminal kinases (JNK) phosphorylates S63 and S73 of c-Jun, a component of the AP-1 transcription factor crucial in regulating cell cycle progression and apoptosis, while activation of PKC results in dephosphorylation of inhibitory residues on AP-1 [49,59]. Interestingly, JNK function is required for Cdk4 upregulation in cellular systems with NF-κB downregulation, a component of squamous cell carcinomas [60], suggesting that JNK, Cdk4, and PKC may comprise a regulatory loop crucial in promoting neurogenesis and pro-survival signaling. Whether these kinases form a separate cluster will require further investigations, but data from the literature indicate that this might be a possibility. Other kinases identified in our study may also control FGF14:Nav1.6 channel complementation. The NF-κB pathway has been implicated in neuronal plasticity, survival, and injury [61,62], and components of the NF-κB pathway have been found to be expressed at the AIS [63]. The NF-κB inhibitors BAY 11–7082 and BAY 11–7085 potently inhibit FGF14:Nav1.6 complementation, consistent with earlier results [26], suggesting that NF-κB activation may be important in promoting neuronal excitability through promotion of AIS complex formation. Additionally, eEF2K may regulate dendritic spine stability and morphology through activity-dependent dendritic BDNF synthesis [64], and defects in eEF2K impairs associative taste learning and produces abnormal brain area activation in mice [65]. Both NH125 and TX-1918, potent inhibitors of eEF2K, induce strong disruption of FGF14:Nav1.6 interaction at higher doses. Furthermore NH125 appears to produce a bimodal dose-response curve, suggesting a complex dependence may exist between eEF2K activity and neuronal excitability. Interestingly, lithium, in addition to its role as a GSK-3 inhibitor, upregulates elongation factor 2 (eEF2) through prevention of inhibitory phosphorylation at Thr56, and direct GSK-3 inhibition unexpectedly leads to an enhancement of eEF2 phosphorylation [66], suggesting that both GSK-3 and eEF2K may play opposing roles in regulating eEF2 activity.

We were the first to adapt the split-luciferase assay for studying protein:channel interactions in live cells [25,26] and here we complemented this powerful methodology with an array of dose-response studies and bioinformatics tools that gave us a unique approach for discovering new signaling pathways in a rapid and efficient way. The efficacy of kinase inhibition, especially in *ex vivo* and *in vivo* systems, is highly heterogeneous and depends on a variety of chemical factors such as solubility and lipophilicity, as well as biological factors such as off-target effects and toxicity. These observations highlight the difficulty of obtaining selective inhibition of the desired target [67,68], and have spurred efforts to profile inhibitor selectivity against the comprehensive kinome [31]. As such, investigating the dose-response characteristics of compounds that pass the initial screening, as well as the minimum active dose required for efficacy, facilitates investigation of FGF14:Nav complementation while minimizing the impact of off-target effects. Additionally, research into kinase signaling pathways has revealed that many physiological systems are controlled by bimodal or multiphasic response profiles [69–71], complicating drug discovery [72] and highlighting the need to understand dose-response profiles.

Through validation of an innovative bioluminescence-based screening of ∼400 kinase inhibitors, we have identified multiple pathways that might be directed through the tyrosine kinase/PI3K/Akt regulatory pathway as well as Wee1 kinase and PKC that, independently or through modulatory feedback loops, modifies FGF14:Nav1.6 complementation. We have additionally shown that these regulatory pathways are part of a GSK3-centered signaling network and that inhibition of GSK3 suppresses neuronal excitability. In light of the significance of GSK3 in psychiatric disorders, these data reinforce the importance of GSK3 in organization of the Nav channel complex as well as modulation of neuronal activity. These novel results serve as the first step towards constructing a functional interaction network of excitability, which broadens the targets available for therapeutic interventions against brain disorders and provides a deeper understanding of the pathogenesis of these disorders. Further studies must expand upon these interactions to determine the specifics of interdependent regulatory networks that may regulate neuronal excitability.

The management of complex brain disorders requires new paradigms of data collection, analysis, and synthesis [73–75]. Through a combination of high-throughput screening techniques, detailed dose-response validation, and innovative bioinformatics processing of interaction strength data, our approach delivers new insights into complex regulatory pathways that may underlie the pathogenesis of these recalcitrant diseases. Additionally, by identifying a key list of compounds believed to influence protein:protein interactions at the AIS, and subsequently neuronal excitability, our results provide both an opportunity to discover novel uses for existing commercial inhibitors, and a benchmark for conducting structure-based drug discovery projects to design more specific and more potent inhibitors against these relevant kinases.

## Supporting Information

S1 Table Details and source of kinase inhibitors used in the study. From left: primary kinase target of the inhibitor, name of the inhibitor, reported *in vitro* IC50 values in literature, company from where the inhibitor was procured, mechanism of action with regard to ATP competitiveness, literature reference for IC50 value.
(DOCX)
