# Supplementary material for: Identifying a Kinase Network Regulating FGF14:Nav1.6 Complex Assembly Using Split-Luciferase Complementation
Source: PLoS One. 2015 Feb 6;10(2):e0117246. doi: 10.1371/journal.pone.0117246 (PMC4319734; doi:10.1371/journal.pone.0117246)
Supplement: S1 Table — From left: primary kinase target of the inhibitor, name of the inhibitor, reported in vitro IC50 values in literature, company from where the inhibitor was procured, mechanism of action with regard to ATP competitiveness, literature reference for IC50 value. (DOCX) [file pone.0117246.s001.docx]

| **Kinase** | **Inhibitor** | **IC50 (nM)** | **Company** | **Mechanism** | **Reference** |
| --- | --- | --- | --- | --- | --- |
| Akt | Triciribine | 130 | Tocris | Non-ATP competitive | [1] |
| Akt | GSK690693 | 13 | Selleckchem | ATP-competitive | [2] |
| Cdk1 | Cdk1 inhibitor | 5800 | Santa Cruz Biotech | ATP-competitive | [3] |
| Cdk1 | Ro 3306 | 20 | Santa Cruz Biotech | ATP-competitive | [4] |
| Cdk4 | Cdk4 inhibitor | 76 | EMD Millipore | ATP-competitive | [5] |
| Cdk4 | NSC 625987 | 200 | Tocris | ATP-competitive | [6] |
| eEF2K | NH125 | 60 | Cayman Chemical | Non-ATP competitive | [7] |
| eEF2K | TX-1918 | 440 | Santa Cruz Biotech | Non-ATP competitive | [8] |
| JNK | JNK inhibitor IX | 316 | Tocris | ATP-competitive | [9] |
| JNK | JNK inhibitor XVI | 18.7 | EMD Millipore | Non-ATP competitive | [10] |
| MLCK | ML-7 hydrochloride | 300 | Tocris | ATP-competitive | [11] |
| MLCK | MLCK Inhibitor Peptide 18 | 50 | Tocris | Mixed-ATP-competitive | [12] |
| NF-kB | BAY11–7085 | 10000 | Santa Cruz Biotech | Non-ATP competitive | [13] |
| NF-kB | BAY11–7082 | 10000 | EMD Millipore | Non-ATP competitive | [13] |
| PIKfyve | YM201636 | 33 | Cayman Chemical | ATP-competitive | [14] |
| PI3K | LY294002 | 500 | Cayman Chemical | ATP-competitive | [15] |
| PI3K | Wortmannin | 2 | Cayman Chemical | ATP-competitive | [16] |
| PKC | Go 6983 | 7 | Tocris | ATP-competitive | [17] |
| PKC | Go 6976 | 7.9 | Tocris | ATP-competitive | [18] |
| Syk | Syk inhibitor III | 2500 | Tocris | Unknown | [19] |
| Syk | Syk Inhibitor IV | 7.5 | Santa Cruz Biotech | ATP-competitive | [20] |
| Wee1 | Wee1 inhibitor | 11 | EMD Millipore | ATP-competitive | [21] |
| Wee1 | Wee1 inhibitor II | 59 | EMD Millipore | ATP-competitive | [21] |

**Table S1:**

1. Gursel DB, Connell-Albert YS, Tuskan RG, Anastassiadis T, Walrath JC, et al. (2011) Control of proliferation in astrocytoma cells by the receptor tyrosine kinase/PI3K/AKT signaling axis and the use of PI-103 and TCN as potential anti-astrocytoma therapies. Neuro Oncol 13: 610-621.

2. Rhodes N, Heerding DA, Duckett DR, Eberwein DJ, Knick VB, et al. (2008) Characterization of an Akt kinase inhibitor with potent pharmacodynamic and antitumor activity. Cancer Res 68: 2366-2374.

3. Fedorov O, Marsden B, Pogacic V, Rellos P, Muller S, et al. (2007) A systematic interaction map of validated kinase inhibitors with Ser/Thr kinases. Proc Natl Acad Sci U S A 104: 20523-20528.

4. Vassilev LT, Tovar C, Chen S, Knezevic D, Zhao X, et al. (2006) Selective small-molecule inhibitor reveals critical mitotic functions of human CDK1. Proc Natl Acad Sci U S A 103: 10660-10665.

5. Zhu G, Conner SE, Zhou X, Shih C, Li T, et al. (2003) Synthesis, structure-activity relationship, and biological studies of indolocarbazoles as potent cyclin D1-CDK4 inhibitors. J Med Chem 46: 2027-2030.

6. Kubo A, Nakagawa K, Varma RK, Conrad NK, Cheng JQ, et al. (1999) The p16 status of tumor cell lines identifies small molecule inhibitors specific for cyclin-dependent kinase 4. Clin Cancer Res 5: 4279-4286.

7. Yamamoto K, Kitayama T, Ishida N, Watanabe T, Tanabe H, et al. (2000) Identification and characterization of a potent antibacterial agent, NH125 against drug-resistant bacteria. Biosci Biotechnol Biochem 64: 919-923.

8. Hori H, Nagasawa H, Ishibashi M, Uto Y, Hirata A, et al. (2002) TX-1123: an antitumor 2-hydroxyarylidene-4-cyclopentene-1,3-dione as a protein tyrosine kinase inhibitor having low mitochondrial toxicity. Bioorg Med Chem 10: 3257-3265.

9. Angell RM, Atkinson FL, Brown MJ, Chuang TT, Christopher JA, et al. (2007) N-(3-Cyano-4,5,6,7-tetrahydro-1-benzothien-2-yl)amides as potent, selective, inhibitors of JNK2 and JNK3. Bioorg Med Chem Lett 17: 1296-1301.

10. Zhang T, Inesta-Vaquera F, Niepel M, Zhang J, Ficarro SB, et al. (2012) Discovery of potent and selective covalent inhibitors of JNK. Chem Biol 19: 140-154.

11. Saitoh M, Ishikawa T, Matsushima S, Naka M, Hidaka H (1987) Selective inhibition of catalytic activity of smooth muscle myosin light chain kinase. J Biol Chem 262: 7796-7801.

12. Lukas TJ, Mirzoeva S, Slomczynska U, Watterson DM (1999) Identification of novel classes of protein kinase inhibitors using combinatorial peptide chemistry based on functional genomics knowledge. J Med Chem 42: 910-919.

13. Pierce JW, Schoenleber R, Jesmok G, Best J, Moore SA, et al. (1997) Novel inhibitors of cytokine-induced IkappaBalpha phosphorylation and endothelial cell adhesion molecule expression show anti-inflammatory effects in vivo. J Biol Chem 272: 21096-21103.

14. Jefferies HB, Cooke FT, Jat P, Boucheron C, Koizumi T, et al. (2008) A selective PIKfyve inhibitor blocks PtdIns(3,5)P(2) production and disrupts endomembrane transport and retroviral budding. EMBO Rep 9: 164-170.

15. Chaussade C, Rewcastle GW, Kendall JD, Denny WA, Cho K, et al. (2007) Evidence for functional redundancy of class IA PI3K isoforms in insulin signalling. Biochem J 404: 449-458.

16. Arcaro A, Wymann MP (1993) Wortmannin is a potent phosphatidylinositol 3-kinase inhibitor: the role of phosphatidylinositol 3,4,5-trisphosphate in neutrophil responses. Biochem J 296 ( Pt 2): 297-301.

17. Gschwendt M, Dieterich S, Rennecke J, Kittstein W, Mueller HJ, et al. (1996) Inhibition of protein kinase C mu by various inhibitors. Differentiation from protein kinase c isoenzymes. FEBS Lett 392: 77-80.

18. Martiny-Baron G, Kazanietz MG, Mischak H, Blumberg PM, Kochs G, et al. (1993) Selective inhibition of protein kinase C isozymes by the indolocarbazole Go 6976. J Biol Chem 268: 9194-9197.

19. Wang WY, Wu YC, Wu CC (2006) Prevention of platelet glycoprotein IIb/IIIa activation by 3,4-methylenedioxy-beta-nitrostyrene, a novel tyrosine kinase inhibitor. Mol Pharmacol 70: 1380-1389.

20. Yamamoto N, Takeshita K, Shichijo M, Kokubo T, Sato M, et al. (2003) The orally available spleen tyrosine kinase inhibitor 2-[7-(3,4-dimethoxyphenyl)-imidazo[1,2-c]pyrimidin-5-ylamino]nicotinamide dihydrochloride (BAY 61-3606) blocks antigen-induced airway inflammation in rodents. J Pharmacol Exp Ther 306: 1174-1181.

21. Palmer BD, Thompson AM, Booth RJ, Dobrusin EM, Kraker AJ, et al. (2006) 4-Phenylpyrrolo[3,4-c]carbazole-1,3(2H,6H)-dione inhibitors of the checkpoint kinase Wee1. Structure-activity relationships for chromophore modification and phenyl ring substitution. J Med Chem 49: 4896-4911.
